# Should face-to-face in-person therapy be preserved for some clients with anxiety? Evaluation of Anxiety UK's psychological therapy services before and during the COVID-19 pandemic

**DOI:** 10.1192/bjo.2024.738

**Published:** 2024-10-25

**Authors:** Lewis W. Paton, Penny Bee, Kate Bosanquet, Peter Bower, Jason Fell, Judith Gellatly, Nicky Lidbetter, Beatrice Lukoseviciute, Dean McMillan, Dave Smithson, Paul A. Tiffin

**Affiliations:** Hull York Medical School, University of York, York, UK; Department of Health Sciences, University of York, York, UK; School of Health Sciences, University of Manchester, Manchester, UK; Anxiety UK, Manchester, UK

**Keywords:** Anxiety, psychological therapy, COVID-19, remote, face-to-face

## Abstract

**Background:**

The COVID-19 pandemic initiated a mass switch to psychological therapy being delivered remotely, including at Anxiety UK, a national mental health charity. Understanding the impact of this forced switch could raise implications for the provision of psychological therapies going forwards.

**Aims:**

To understand whether the forced switch to remote therapy had any impact on outcomes, and if certain groups should continue to be routinely offered certain delivery modalities in future.

**Method:**

Data were available for 2323 individuals who accessed Anxiety UK services between January 2019 and October 2021. Demographic data, baseline and discharge anxiety and depression symptoms, and mode of therapy delivery were available.

Regression models were built to model (a) the mode of therapy delivery received pre-pandemic using logistic regression, and (b) outcomes pre- and post-pandemic onset within demographic groups.

**Results:**

No statistically significant changes in baseline anxiety symptoms, demographics or outcomes were observed before and after the onset of the COVID-19 pandemic.

Pre-pandemic, males were more likely to receive online video therapy than telephone therapy (Relative Risk Ratio (RRR) 1.42, [1.01, 1.99]), while older clients were less likely to receive online video therapy (RRR 0.98, [0.97, 0.99]). However, no differences in outcomes were observed post-pandemic onset within these groups, with only the number of sessions of therapy being a significant predictor of outcomes.

**Conclusions:**

Anxiety UK services remained effective throughout the pandemic. We observed no evidence that any demographic group had worse outcomes following the forced switch to remote therapy.

The COVID-19 pandemic was a significant worldwide threat to health, and had major impacts on mental health, especially depression and anxiety.^1–3^ Equally, the pandemic and its associated impacts (such as lockdowns and social isolation) led to equally profound changes in health service delivery, including the delivery of mental health and psychological therapy services, with a major change being a wholesale shift to remote delivery (through telephone, online video or other digital means) to reduce infection risks.^4^

Although the relationship between the client and therapist is generally seen as a key ‘active ingredient’ of effective psychological therapy,^5^ comparable alliance ratings can be achieved remotely and face-to-face,^6^ and remote delivery has a long history in the field. This was driven by the need to provide access to certain populations (such as rural areas) and to improve the efficiency and cost-effectiveness of treatment. Reviews have generally indicated that such services can be effective and acceptable, although with potentially additional complications for both patient and therapist.^6–11^

In England, UK, a key service for the management of depression and anxiety is the National Health Service (NHS) Talking Therapies services. Until 2023 this service was known as the Improving Access to Psychological Therapies (IAPT) programme,^12–14^ and we refer to IAPT in this study for consistency with the literature at that time. IAPT supports the implementation of a stepped-care approach,^14^ with the provision of psychological treatment being offered in the most effective and efficient way. It aims to rebalance resources across the patient treatment pathway, providing an increasingly high volume of people with evidence-based treatment without compromising clinical outcomes and cost-effectiveness.^15^ The least intensive treatments (in terms of treatment intensity, practitioner time and cost) likely to provide significant health gain are offered in the first instance.^16^ The model is ‘self-correcting’ where a systematic process of monitoring treatment outcome is conducted. Where initial treatment provides no significant gain (determined through the administration of outcome measures), more intensive options are explored. Step 1 consists of identification and assessment of known or suspected presentations. Step 2 consists of low-intensity therapies (for example, guided self-help), often led by psychological well-being practitioners. Individuals who do not respond, or who have more severe or complex symptoms, will be offered higher-intensity interventions (for example, cognitive-behavioural therapy) at Step 3. Highly specialised treatment, including crisis services or in-patient care, is offered at Step 4.^14^

Although remote delivery of psychological therapy is common in IAPT, particularly at lower steps, pre-pandemic there was significant variability in the use of remote delivery between IAPT services and between different steps of the ‘stepped-care’ service model, with use at Step 2 most common. However, following the pandemic there was a sudden change to delivery such that all delivery was remote. Published studies have begun to explore these issues and their effects on patients and professionals. Early evidence suggested an initial reduction in overall IAPT referrals at the onset of the pandemic, and an additional increase in severity of referrals.^17^ No worsening of outcomes during remote provision compared with 2019 was observed in two IAPT services, and indeed some groups appeared to benefit from the switch to remote therapy.^18^ Other IAPT services reported no difference in improvement in symptoms overall following the switch to remote services, but did report a rapid decrease in patient symptoms.^19^ On a group level, therefore, the emerging evidence is that the switch to remote therapy as a consequence of the pandemic had no detrimental impact on patient outcomes. However, it remains unclear as to whether there are certain individuals for whom a forced switch to remote services would have a negative effect.

Pre-pandemic, individuals received face-to-face or remote services depending on a range of factors, including their local IAPT service configuration, their personal characteristics and preferences, and the clinical decisions of local professionals. Post-pandemic, all people received remote services. IAPT has a strong culture of outcome measurement, and throughout this period people moving through the service had their outcomes measured throughout their treatment. The sudden effect of the COVID-19 pandemic and the resulting public health measures had potentially negative effects on clients of IAPT services, particularly given the observed benefits of incorporating patient preferences on outcomes.^20^ However, it did create an opportunity to evaluate the possible impacts of such changes. Our findings could thus allow patients, professionals and policymakers to better prepare for the future.

Anxiety UK^21^ is a national mental health charity which offers a range of support and information services for those living with, and affected by, anxiety disorders, including anxiety-based depression. Clients present for support with a wide range of anxiety conditions, as well as obsessive–compulsive disorder, of varying intensity and severity. While Anxiety UK does not operate a stepped-care model, the charity's psychological therapy service offers interventions that equate to Steps 2 and 3 of the IAPT stepped-care model. Individuals can access IAPT or Anxiety UK services, although it would not be recommended that two therapy interactions be undertaken concurrently. Anxiety UK's therapy services are chargeable, with fees based on income, and the number of sessions provided depends on the therapy type and individual client.^22^ Inclusion and exclusion criteria are provided in Fig. 1.

**Fig. 1:**
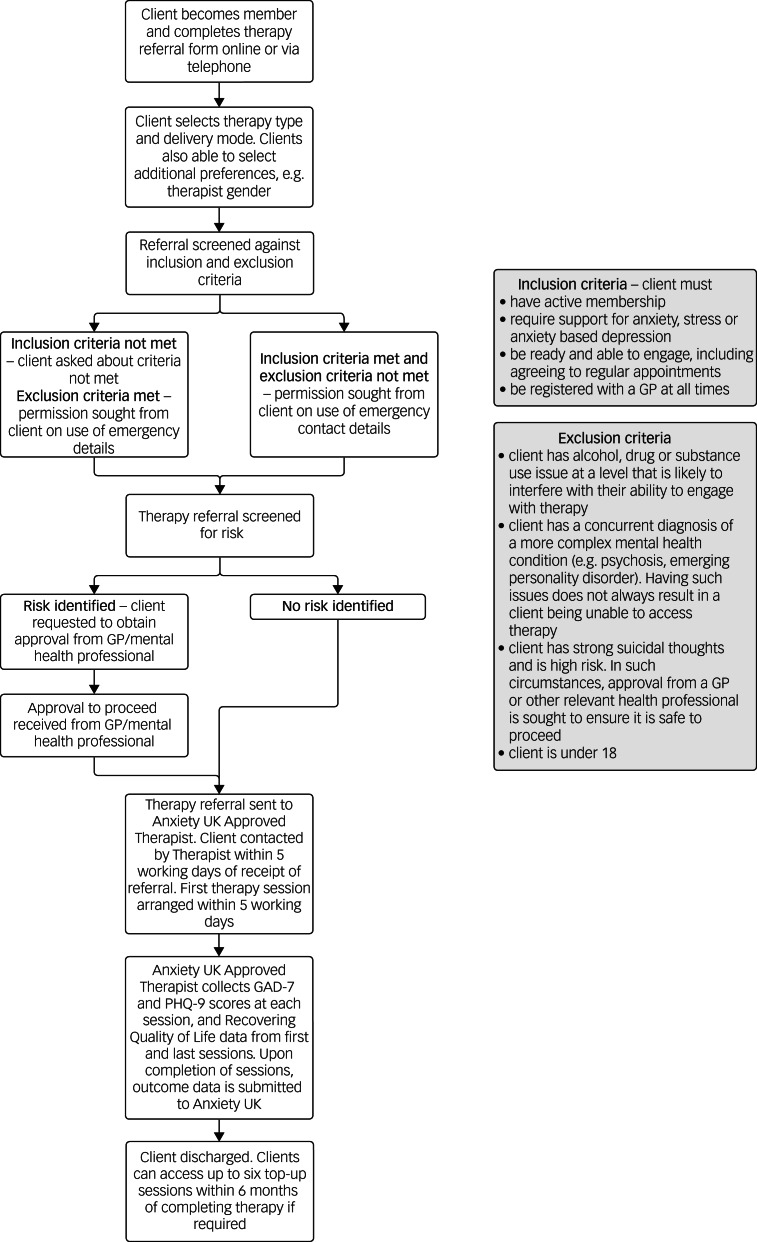
Anxiety UK referral and therapy process, including inclusion and exclusion criteria. GP, general practitioner; GAD-7, Generalised Anxiety Disorder-7; PHQ-9, Patient Health Questionnaire-9.

Clients self-refer or access the service following a referral from a partner agency, usually charitable benevolent organisations (see Fig. 1), where a range of non-pharmacological therapies are made available. Therapy modalities provided include Cognitive Behavioural Therapy, counselling, Compassion Focused Therapy, Eye Movement Desensitisation and Reprocessing, and clinical hypnotherapy, in addition to a number of self-help solutions. Therapy sessions are offered at affordable rates based on household income and are delivered through the charity's network of Anxiety UK Approved Therapists via telephone, online via webcam, and in non-pandemic times, in person.

Previous research has shown that outcomes for clients accessing Anxiety UK services exceeds IAPT targets for outcomes,^23^ with a similar reduction in anxiety and depression seen across various delivery modes (telephone, online and face-to-face), demonstrating that the charity provides effective psychological therapies for anxiety and depression.

In this paper, we use data from Anxiety UK collected before and after the onset of the COVID-19 pandemic. We use these data to explore both the overall impact of the pandemic and the shift to remote delivery. We therefore seek to understand whether the impacts of that shift were greater on clients who, pre-pandemic, would have been more likely to receive face-to-face services. Specifically, we aimed to:

- Understand whether client profiles changed after the onset of the COVID-19 pandemic
- Understand the potential impact of remote delivery of services on client outcomes during the pandemic
- Explore whether, pre-pandemic, there was a relationship between therapy delivery mechanism and baseline demographic or clinical variables
- If differences to (c) were observed, to explore, when all services were remote, whether those groups more likely to have received face-to-face therapy pre-pandemic had worse outcomes during the pandemic

## Method

### Data availability

Data were available for all 2323 individuals who accessed Anxiety UK psychological therapy services between January 2019 and October 2021. Of these, 180 had at least two separate courses of therapy. We included data only from the first course of therapy.

For the purposes of analysis, we treated 23 March 2020, the date of the start of the first COVID-19 lockdown in the UK, as the ‘start’ of the COVID-19 pandemic. When considering analyses regarding baseline information on clients, they were dichotomised into those who:

- Started their therapy before 23 March 2020, and
- Started on or after this date.

Similarly, when considering analyses relating to outcomes of therapy, clients could be trichotomised into:

- Those who started and finished their therapy before the onset of the pandemic
- Those who started therapy before the onset of the pandemic, but finished after 23 March 2020, and
- Those who started and finished after the onset of the pandemic.

### Variables

#### Therapy details

For each client, we had access to their start and end date of therapy, and the number of sessions of therapy received. Data were available on the modality of psychological therapy received, namely Cognitive Behavioural Therapy, clinical hypnotherapy or counselling. Additionally, we had data on how the therapy was delivered: face-to-face, telephone, video or a mixed approach (‘blended’).

Clients self-complete the Generalised Anxiety Disorder Scale-7 (GAD-7)^24^ and the Patient Health Questionnaire-9 (PHQ-9)^25^ as a measure of depression severity. We had access to the scores on the first and last session of therapy. ‘Caseness’ was defined as PHQ-9 score of 10 or more, or GAD-7 score of 8 or higher.^26^ From this, three outcomes for therapy can be defined, in line with NHS IAPT definitions.^27^ Specifically:

- Recovery: where a client reported symptom scores exceeding those required for caseness at the beginning of therapy, but both PHQ-9 and GAD-7 were below the caseness threshold at the end of therapy.
- Reliable improvement: a reduction in GAD-7 and/or PHQ-9 that exceeded the measurement error of the scale. Specifically, a reduction of four points for GAD-7, and six for PHQ-9.
- Reliable recovery: both recovery and reliable improvement criteria were met.

#### Sociodemographic variables

For each participant, self-reported data were available on age at the start of therapy, gender, sexuality (dichotomised into heterosexual or Lesbian, Gay, Bisexual, Transgender or other (LGBT+)) and ethnicity (dichotomised into White or Black and minority ethnic).

### Statistical analysis

All data were analysed using Stata version 17 (for Windows).^28^ To answer the aims of the paper, we performed the following specific analyses. Given that we were interested in the delivery mechanism of therapy, we made no distinction in our analyses between the different types of therapy received. Given the limited demographic data, missing data were handled using listwise deletion.

#### Aim 1: to understand whether client profiles changed after the onset of the COVID-19 pandemic

Differences in demographic variables were assessed pre and post the onset of the COVID-19 pandemic using Mann–Whitney *U* testing. Linear regression analyses were performed to understand if baseline anxiety symptoms changed before and after the onset of the pandemic. Univariable and multivariable regression analyses were performed to understand inter-group differences in baseline anxiety symptoms.

#### Aim 2: to understand the potential impact of remote delivery of services on client outcomes during the COVID-19 pandemic

Logistic regression models were built for each outcome variable. We used an indicator variable for the trichotomised time period (started and finished pre-pandemic, started pre-pandemic and finished post-pandemic, started and finished post-pandemic) to assess differences in outcomes. Multivariable models were then built controlling for the number of sessions and demographic variables. We restricted analyses to those who had received at least two sessions of therapy.

#### Aim 3: to explore whether, pre-pandemic, there was a relationship between therapy delivery mechanism and baseline demographic or clinical variables

For those who started their therapy before the onset of the COVID-19 pandemic, a series of multinomial logistic regression models were used to estimate the relationship between baseline variables and delivery mechanism received. Univariable models were initially built. Any statistically significant variables on univariable analysis were then included in a multivariable model. The relationship between the continuous age variable and delivery mechanism was modelled by multivariable fractional polynomials.

#### Aim 4: to explore, when all services were remote, whether those groups more likely to have received face-to-face therapy pre-pandemic had worse outcomes during the COVID-19 pandemic

In those clients who started therapy after the onset of the COVID-19 pandemic, logistic regression models were built for each outcome variable (*recovery, reliable improvement* and *reliable recovery*). We included as covariates those variables, if any, which were significantly associated with delivery choice in those who started therapy *before* the onset of the COVID-19 pandemic.

## Results

### Aim 1: to understand whether client profiles changed after the onset of the COVID-19 pandemic

Of our 2323 included clients, 1480 (63.7%) started before 23 March 2020, and 843 afterwards. No differences were observed across demographic groups (Table 1). On univariable linear regression analysis, whether a client had started before the onset of the COVID-19 pandemic was statistically non-significant when predicting baseline GAD-7 scores (*β* = −0.04, −0.44 to 0.37, *P* = 0.86). That is, GAD-7 scores were, on average, 0.04 lower for those who started therapy before the onset of the pandemic than for those who started afterwards, but this was not statistically significant from zero change at the *P* = 0.05 level. Similarly, whether a client started therapy before the onset of the pandemic was not a statistically significant predictor of baseline PHQ-9 scores (*β* = 0.33, −0.18 to 0.84, *P* = 0.20).

**Table 1:** Demographic data, split by whether clients started therapy before or after the onset of the COVID-19 pandemic. Note that one individual who started therapy post-pandemic was recorded as receiving counselling and clinical hypnotherapy, and another as counselling and compassion-focused therapy. These two individuals are included in the ‘counselling’ figure.

	Started therapy before the onset of the pandemic (*n* = 1480)	Started therapy after the onset of the pandemic (*n* = 843)
Male (versus female)	556/1477 (37.6%)	326/843 (38.7%)
LGBT+ sexuality	96/944 (10.2%)	36/232 (15.5%)
Black or minority ethnic	95/699 (13.6%)	64/439 (14.6%)
Age (mean, s.d.)	36.5 (12.6)	34.1 (12.9)
Baseline GAD-7 score (mean, s.d.)	13.2 (4.71)	13.2 (4.69)
Baseline PHQ-9 score (mean, s.d.)	12.4 (5.99)	12.0 (5.80)
Delivery mechanism		
Face-to-face	747/1480 (50.5%)	
Telephone	289/1480 (19.5%)	156/843 (18.5%)
Video	300/1480 (20.3%)	684/843 (81.1%)
Blended	144/1480 (9.7%)	3/843 (0.36%)
Therapy type		
Cognitive Behavioural Therapy	721/1480 (48.7%)	466/843 (55.3%)
Clinical hypnotherapy	229/1480 (15.5%)	61/843 (7.24%)
Counselling	530/1480 (35.8%)	316/843 (37.5%)

LGBT+, Lesbian, Gay, Bisexual, Transgender or other (+); GAD-7, Generalised Anxiety Disorder-7; PHQ-9, Patient Health Questionnaire-9.

On multivariable analysis, both male gender (*β* = −0.55, −0.87 to −0.24, *P* = 0.01) and baseline PHQ-9 score (*β* = 0.49, 0.46 to 0.52, *P* < 0.001) were statistically significant predictors of baseline GAD-7 symptoms, controlling for the period a client started therapy (*β* = −0.21, −0.52 to 0.11, *P* = 0.21).

### Aim 2: to understand the impact of remote delivery of services on client outcomes during the COVID-19 pandemic

Of our 2323 included clients, 1219 started and finished before the onset of the COVID-19 period, 261 started before the onset of the pandemic but finished after, and 843 started after the pandemic. Of these, 1144 (93.8%), 258 (98.9%) and 803 (95.3%) respectively attended at least two sessions of therapy.

Table 2 displays the proportion of clients who achieved each outcome, as well as the number of sessions, for each of the three time periods. As can be seen, outcome proportions appeared reasonably consistent across the three time periods considered. One exception appeared to be ‘reliable improvement’ in the period where clients straddled the onset of the COVID-19 pandemic. In this case, reliable improvement appeared somewhat higher. On univariable logistic regression modelling, those who started therapy before the onset of the pandemic but finished afterwards had statistically significantly higher odds of fulfilling the reliable improvement criteria than those in time period 1 (odds ratio 1.44, 1.05 to 1.98, *P* = 0.02). However, after controlling for the potential influence of the number of sessions attended, this difference becomes non-significant. No other tested differences were statistically significantly different.

**Table 2:** Descriptive data on number of sessions, and clinical outcomes, stratified by when clients started and finished their therapy in relation to the COVID-19 pandemic

	Started and finished therapy before the onset of the pandemic	Started therapy before the onset of the pandemic, and finished therapy after	Started and finished therapy after the onset of the pandemic
	(*n* = 1144)	(*n* = 258)	(*n* = 803)
Number of sessions	8.32 (4.59)	12.7 (5.35)	10.6 (5.41)
Recovery	602/999 (60.3%)	154/239 (64.4%)	446/731 (61.0%)
Reliable improvement	776/1115 (69.6%)	198/258 (76.7%)	573/801 (71.5%)
Reliable recovery	558/1001 (55.7%)	148/240 (61.7%)	420/731 (57.5%)

### Aim 3: to explore whether, pre-pandemic, there was a relationship between therapy delivery mechanism and baseline demographic or clinical variables

For those who started before the pandemic (*n* = 1480), and thus could receive therapy in a variety of ways, 747 received face-to-face therapy, 300 received video therapy and 289 received telephone therapy. We excluded those who were recorded as receiving ‘blended’ therapy, as we could not distinguish between those who actively chose a blended approach and those who received a blended approach due to having to switch as a result of the COVID-19 pandemic.

As can be seen in Table 3, we did not observe any statistically significant predictors for receiving either of the remote options in comparison with face-to-face therapy. However, there are some differences between the two remote options, with males having higher odds of receiving video therapy in comparison to telephone therapy (Relative Risk Ratio (RRR) 1.42, 1.01 to 1.99, *P* = 0.04). Those with higher baseline GAD-7 scores were less likely to receive video therapy than telephone therapy (RRR 0.85, 0.91 to 0.98, *P* = 0.002), as were those with higher baseline PHQ-9 scores (RRR 0.97, 0.94 to 1.00, *P* = 0.02). Multivariable fractional polynomials suggested a linear relationship between age and delivery. Older clients were less likely to receive video therapy than telephone therapy (RRR 0.98, 0.97 to 0.99, *P* = 0.004).

**Table 3:** Results from univariable multinomial logistic regression models predicting choice of therapy delivery mechanism for those who started their therapy before the onset of the COVID-19 pandemic

	Video versus face-to-face	Telephone versus face-to-face	Video versus telephone
	(RRR, 95% CI, *P*-value)	(RRR, 95% CI, *P*-value)	(RRR, 95% CI, *P*-value)
Male	1.10	0.77	1.42
	(0.84 to 1.45)	(0.58 to 1.03)	(1.01 to 1.99)
	*P* = 0.50	*P* = 0.08	*P* = 0.04
LGBT+ sexuality	0.75	0.88	0.85
	(0.40 to 1.38)	(0.50 to 1.54)	(0.41 to 1.77)
	*P* = 0.35	*P* = 0.65	*P* = 0.67
Black or minority ethnic	0.72	0.56	1.29
	(0.40 to 1.30)	(0.27 to 1.13)	(0.56 to 2.97)
	*P* = 0.27	*P* = 0.10	*P* = 0.55
Baseline GAD-7 score	0.97	1.03	0.85
	(0.95 to 1.00)	(1.00 to 1.06)	(0.91 to 0.98)
	*P* = 0.07	*P* = 0.06	*P* = 0.002
Baseline PHQ-9 score	0.98	1.01	0.97
	(0.96 to 1.01)	(0.99 to 1.04)	(0.94 to 1.00)
	*P* = 0.17	*P* = 0.18	*P* = 0.02
Age	0.99	1.01	0.98
	(0.98 to 1.00)	(1.00 to 1.02)	(0.97 to 0.99)
	*P* = 0.10	*P* = 0.07	*P* = 0.004

CI, confidence interval; LGBT+, Lesbian, Gay, Bisexual, Transgender or Other (+); GAD-7, Generalised Anxiety Disorder-7; PHQ-9, Patient Health Questionnaire-9; RRR, Relative Risk Ratio.

Results from the multivariable analysis are displayed in Table 4. On multivariable analysis, the relationship between baseline symptoms and therapy received became statistically non-significant. Furthermore, controlling for gender and baseline symptoms, older clients were more likely to receive telephone therapy than face-to-face therapy (RRR 1.01, 1.00 to 1.02, *P* = 0.04), and less likely to receive video instead of telephone (RRR 0.98, 0.97 to 0.99, *P* = 0.01). There was a trend for males to be less likely to receive therapy via telephone instead of face-to-face, although this was not statistically significant at the *P* = 0.05 level (RRR 0.75, 0.56 to 1.01, *P* = 0.06).

**Table 4:** Results from multivariable multinomial logistic regression models predicting delivery mechanism for therapy in those who started their therapy before the onset of the COVID-19 pandemic

	Video versus face-to-face	Telephone versus face-to-face	Video versus telephone
	(RRR, 95% CI, *P*-value)	(RRR, 95% CI, *P*-value)	(RRR, 95% CI, *P*-value)
Male	1.10	0.75	1.46
	(0.83 to 1.45)	(0.56 to 1.01)	(1.03 to 2.07)
	*P* = 0.52	*P* = 0.06	*P* = 0.04
Baseline GAD-7 score	0.97	1.02	0.96
	(0.94 to 1.01)	(0.98 to 1.06)	(0.91 to 1.00)
	*P* = 0.24	*P* = 0.26	*P* = 0.05
Baseline PHQ-9 score	1.00	1.00	0.99
	(0.97 to 1.03)	(0.97 to 1.03)	(0.96 to 1.03)
	*P* = 0.88	*P* = 0.81	*P* = 0.74
Age	0.99	1.01	0.98
	(0.98 to 1.00)	(1.00 to 1.02)	(0.97 to 0.99)
	*P* = 0.18	*P* = 0.04	*P* = 0.01

CI, confidence interval; LGBT+, Lesbian, Gay, Bisexual, Transgender or other (+); GAD-7, Generalised Anxiety Disorder-7; PHQ-9, Patient Health Questionnaire-9; RRR, Relative Risk Ratio.

### Aim 4: to explore, when all services were remote, whether those groups more likely to have received face-to-face therapy pre-pandemic had worse outcomes during the COVID-19 pandemic

As can be seen in Table 5, in those who started therapy after 23 March 2020, in multivariable logistic analysis, neither gender nor age were statistically significant predictors of any of the three outcomes considered. When the number of sessions of therapy was also included in the model, it is this variable which is a statistically significant predictor of each of the outcome variables.

**Table 5:** Results from multivariable logistic regression models, for each of the three outcome variables

	Recovery		Reliable improvement		Reliable recovery	
	Model 1	Model 2	Model 1	Model 2	Model 1	Model 2
	(OR, 95% CI, *P*-value)	(OR, 95% CI, *P*-value)	(OR, 95% CI, *P*-value)	(OR, 95% CI, *P*-value)	(OR, 95% CI, *P*-value)	(OR, 95% CI, *P*-value)
Male	1.29	1.29	1.09	1.07	1.29	1.28
	(0.95 to 1.76)	(0.94 to 1.76)	(0.79 to 1.50)	(0.77 to 1.48)	(0.95 to 1.75)	(0.94 to 1.75)
	*P* = 0.11	*P* = 0.12	*P* = 0.60	*P* = 0.68	*P* = 0.10	*P* = 0.11
Age	0.99	1.00	0.99	0.99	0.99	0.99
	(0.98 to 1.01)	(0.98 to 1.01)	(0.98 to 1.00)	(0.98 to 1.01)	(0.98 to 1.00)	(0.98 to 1.01)
	*P* = 0.39	*P* = 0.53	*P* = 0.13	*P* = 0.28	*P* = 0.21	*P* = 0.32
Number of sessions		1.05		1.11		1.06
		(1.02 to 1.08)		(1.07 to 1.15)		(1.03 to 1.09)
		*P* < 0.001		*P* < 0.001		*P* < 0.001

CI, confidence interval; OR, odds ratio.

## Discussion

In this paper we analysed the performance of Anxiety UK's psychological therapy services during the period that included the COVID-19 pandemic and the periods of national ‘lockdown’. Overall, across this period, we observed few differences in demographics, client symptoms or outcomes from therapy. We observed no difference in average baseline anxiety or depressive symptoms, as measured by the GAD-7 and PHQ-9, before and after the onset of the pandemic. Importantly, outcomes from therapy were generally stable across the pandemic period. Additionally, while there were some observable demographic differences in therapy delivery before the onset of the pandemic, there was no statistically significant relationship between the available demographic data and outcomes from therapy following the mass switch to remote therapy. Indeed, only the number of sessions of therapy received was a statistically significant predictor of therapy outcome following the onset of the COVID-19 pandemic.

### Potential interpretations

Our findings suggest that, despite concerns over the impact of the pandemic, outcomes from therapy for those accessing Anxiety UK services were unaffected. Recovery, reliable improvement and reliable recovery rates are broadly in line with those reported in an earlier, pre-pandemic analysis of Anxiety UK's psychological therapy services.^23^ Given that client demographic profiles also remained unchanged across the period studied, our findings are not simply a result of different client symptom profiles. Our results are consistent with some of the existing emerging evidence regarding the lack of impact of the pandemic on outcomes in IAPT services.^18,19^

Our analyses looking at the demographic differences in pre-pandemic therapy delivery and post-pandemic onset therapy outcomes suggest the mode of delivery is of minor importance, if at all relevant, to its effectiveness. We found no evidence that certain groups would benefit from a return to predominantly face-to-face therapy, at least in terms of outcomes. Taken together, these findings suggest that psychological therapies (delivered by the NHS and non-statutory organisations) coped well with the ‘shock’ of the COVID-19 pandemic and the mass switch to remote-only therapy.

Indeed, it was only the number of sessions that predicted outcomes from therapy. This is in line with previous findings from both Anxiety UK and IAPT services.^23,29^ As observed in previous analyses, the average number of sessions of therapy for clients accessing Anxiety UK services is higher than for IAPT patients, which may explain why key performance indicators remain higher for Anxiety UK clients than IAPT patients.^30^ Following on from this, we observed variations in the number of sessions undertaken by clients throughout the study period. The average number of sessions for those who started and finished before the pandemic was 8.32, and rose to 10.6 (for those who started and completed therapy post-pandemic) and 12.7 (for those who started pre-pandemic and completed during the pandemic). These figures provide potential interesting insight into client demand which may be linked to therapy delivery model, severity and complexity of client presentation, as well as the overall impact of the pandemic on mental health in general.

### Implications for practice and policy

While the COVID-19 pandemic may have had an effect on mental health, relatively little changed in terms of outcomes following the onset of the pandemic. This has a number of implications for Anxiety UK and other psychological therapy providers. It is likely that psychological therapy providers such as Anxiety UK will continue to offer a blended model of therapy delivery as occurred before the pandemic, although with greater levels of remote therapy. Indeed, in 2022, a large percentage of referrals to Anxiety UK were for remote therapy, and many Anxiety UK Approved Therapists are opting to continue operating online, potentially because of cost savings, enhanced efficiencies and accessibility.

Our findings suggest that services could continue to be offered remotely with no negative impact on the average client outcomes. That being said, on an individual basis, it is possible that there are certain individuals who would respond better to face-to-face therapy. It has been highlighted that some groups – such as individuals with sensory difficulties or older adults – may be at risk of disadvantage from remote care.^31^ It is also possible that some clients would prefer a certain therapy delivery mechanism even if it is not likely to be more effective for them, in terms of reduction in symptoms. As has been pointed out elsewhere, it may be that some individuals only received therapy because it was remote.^18^ Such client preferences should be considered, where possible.

The number of sessions of therapy was the only statistically significant predictor of outcomes when all clients were receiving remote therapy. As such, ensuring sufficient engagement with therapy is critical to achieving satisfactory outcomes. We could not investigate what ‘sufficient’ means, in the context of remote Anxiety UK services, in this analysis. However, a number of previous studies have investigated factors which are associated with disengagement with therapies,^32,33^ and these should continue to be considered by providers of psychological therapies.

### Strengths and limitations

Our study had a number of strengths. We had a large data-set, providing statistical power to identify differences. Crucially, this data-set spanned both before and after the onset of the COVID-19 pandemic. This provided an opportunity to report on whether clients, symptoms or outcomes changed over the course of the pandemic, providing crucial evidence for service delivery going forward.

However, some limitations must be noted. There were some missing data in the demographics, meaning conclusions should be drawn in light of this. Additionally, limited demographic data and lack of session-by-session data meant that more sophisticated modelling approaches, such as latent modelling or longitudinal modelling, were not feasible. Furthermore, our analysis of delivery mechanisms before the onset of the pandemic relied on analysing the type of therapy actually received. It might be that clients would have preferred a type of therapy that was not available to them – for example, they may have preferred face-to-face therapy, yet could not travel to receive it. We also could not distinguish between those who received a ‘blended’ approach due to a preference to receive therapy over multiple delivery mechanisms, and those who were forced into such a model by the start of the COVID-19 pandemic.

### Directions for future research

On an individual client basis, we had no counterfactual information on what delivery modality of therapy clients would have received during the post-pandemic period, had they had a choice. Consequently, while we cannot draw firm conclusions, given the lack of data, on client preferences of therapy delivery, nevertheless there is some suggestion in our results that, at a group level at least, receiving remote therapy has no detriment on client outcomes, even if there may have been a preference for face-to-face therapy. However, future studies could collect information on client preferences for therapy delivery modality, pre-treatment, in order to model the role of this factor on outcomes, given the evidence for positive benefits on outcomes of incorporating patient preferences in other aspects of therapy.^20^

In this study we did not observe any differences in short-term outcomes across therapy delivery mode for anxiety-related conditions. However, it is possible that, over the medium to longer term, relapse rates do differ by delivery mode. It will thus ideally be important to monitor medium- and long-term outcome rates from Anxiety UK therapies in the post-pandemic world and to monitor drop-out and engagement rates. However, this is, in reality, difficult, as clients do not routinely maintain contact with Anxiety UK beyond the timeframe of their therapy interaction with the organisation. Furthermore, it will be important to consider other technologies for therapy delivery, such as Virtual Reality Therapy.

Overall, the outcomes from Anxiety UK psychological therapy services appeared unaffected by the COVID-19 pandemic, despite the wider effects reported by some psychological therapy services in terms of service delivery and mental health in general. This is supportive of Anxiety UK's overall service delivery model, where online services are central and likely to further expand as new technologies emerge.

## Data Availability

Data used in this study are not publically available. The code used for data cleaning and analysis is available from the corresponding author.
